# Relative Density-Based Intuitionistic Fuzzy SVM for Class Imbalance Learning

**DOI:** 10.3390/e25010034

**Published:** 2022-12-24

**Authors:** Cui Fu, Shuisheng Zhou, Dan Zhang, Li Chen

**Affiliations:** 1 School of Mathematics and Statistics, Xi’dian University, Xi’an 710071, China; 2 School of Computer and Artificial Intelligence, Zhengzhou University, Zhengzhou 450001, China

**Keywords:** fuzzy support vector machine (FSVM), class imbalance learning, intuitionistic fuzzy number (IFN), relative density

## Abstract

The support vector machine (SVM) has been combined with the intuitionistic fuzzy set to suppress the negative impact of noises and outliers in classification. However, it has some inherent defects, resulting in the inaccurate prior distribution estimation for datasets, especially the imbalanced datasets with non-normally distributed data, further reducing the performance of the classification model for imbalance learning. To solve these problems, we propose a novel relative density-based intuitionistic fuzzy support vector machine (RIFSVM) algorithm for imbalanced learning in the presence of noise and outliers. In our proposed algorithm, the relative density, which is estimated by adopting the k-nearest-neighbor distances, is used to calculate the intuitionistic fuzzy numbers. The fuzzy values of the majority class instances are designed by multiplying the score function of the intuitionistic fuzzy number by the imbalance ratio, and the fuzzy values of minority class instances are assigned the intuitionistic fuzzy membership degree. With the help of the strong capture ability of the relative density to prior information and the strong recognition ability of the intuitionistic fuzzy score function to noises and outliers, the proposed RIFSVM not only reduces the influence of class imbalance but also suppresses the impact of noises and outliers, and further improves the classification performance. Experiments on the synthetic and public imbalanced datasets show that our approach has better performance in terms of G-Means, F-Measures, and AUC than the other class imbalance classification algorithms.

## 1. Introduction

The class imbalance learning problem in binary classification occurs when the number of one category is significantly greater than that of the other category [1,2]. The imbalance datasets exist in various application domains, such as biological recognition  [3,4], medical diagnosis [5,6], fault diagnosis [7], credit card fraud detection [8,9], and text categorization [10,11], etc. When tackling imbalanced datasets, due to the main role of the majority class, the traditional classification methods designed for balanced datasets may not always achieve good classification performance for the minority class. Therefore, many improved classical algorithms and novel algorithms [12] have emerged to deal with imbalanced classification. As one of the most classic classification algorithms, SVM [13,14,15] shows relatively more robustness than other methods in imbalanced classification problems, but it is still unsatisfactory. Since the traditional SVM considers all instances equally and ignores the difference between the majority and minority classes, thus the decision surface may be biased toward the majority class instances when handling imbalanced datasets [16,17], especially when there exist noises and outliers.

On the one hand, the fuzzy support vector machine (FSVM) was originally designed to deal with the problem of outliers and noises [18]. The FSVM algorithm uses fuzzy membership functions to assign different fuzzy membership values (MVs) for each instance and redefines the SVM. It allows different instances to make different contributions to the generation of the separated hyperplane. Strategies for calculating fuzzy MVs are very important for the performance of an FSVM method. Currently, the more commonly used method is to give fuzzy value to instances only according to their membership degree of a certain class not the relationship between classes. It results in inaccurate sample distribution information. Based on the intuitionistic fuzzy set [19], Ha and Wang [20] proposed an intuitionistic fuzzy support vector machine (IFSVM), the fuzzy value of which is calculated based on the membership and non-membership degrees determined by the distribution information of the instances. Then, based on intuitionistic fuzzy sets and kernel functions, Ha et al. proposed a new fuzzy support vector machine [21], which is superior in dealing with outliers and noise. Salim et al. [22] introduced the intuitionistic fuzzy number into the twin support vector machine, which evaluated the contribution of instances to the separated hyperplane according to the score function of instances, thus improving the disadvantage that the support vector far from the center of the class is given a lower fuzzy value. However, we know that the contribution of a sample to the classification hyperplane cannot be accurately described only by its distance from the sample center. Moreover, class centers are obtained based on all instances, so outliers may cause class center offset. In addition, the non-membership degree of sparse instances will also be inaccurate due to improper parameter selection.

Many previous works have shown that FSVMs outperform standard SVM in terms of accuracy and robustness, but they are still affected by the imbalanced data distribution. The state-of-the-art techniques to develop the performance of imbalanced data classification are generally called class imbalance learning [23,24] techniques. These techniques can be broadly classified as external and internal. The external techniques deal with the data sets before training a classifier, and the internal techniques create or modify the SVM. We will focus on the latter in this paper. To address the problem of imbalanced datasets classification disturbed by outlier and noise. Batuwita and Palade [24] combine FSVM with cost-sensitive learning to obtain the FSVM-CIL serial algorithms. The six membership functions of it are obtained by combining three different methods with two kinds of decaying functions. Most of them calculate the importance of each instance by Euclidean distance, so the membership functions using this metric are sensitive to the dimension of the data distribution in feature space. Liu [25] modified the distance measure and proposed the Gaussian fuzzy support vector machine (GFSVM), which is an extension of the FSVM-CIL. Experimental results indicated that this improved approach has better performance in class imbalanced learning. Based on the principle of space consistency, Tao et al. presented affinity and class probability-based FSVM (ACFSVM) [26], which can suppress the impact of noises and outliers in the majority class instances on the model. Deep et al. [27] presented an entropy-based entropy-based fuzzy twin support vector machine (EFTWSVM) for imbalanced datasets, which uses the information entropy of instances to determine the membership degree of each instance in the fuzzy twin support vector machine. Richhariya et al. [28] presented a robust fuzzy least squares twin SVM (RFLSTSVM), which employs the 2-norm of the slack variables to make the optimization problem strongly convex and uses the imbalance ratio of instances in calculating the membership degree of samples. Based on the relative density which is a more robust prior information extraction method, Yu et al. [29] presented two fuzzy SVM algorithms to tackle the problem of class imbalance learning, one is the fuzzy SVM algorithm based on the within-class relative density information (FSVM-WD), and the other is fuzzy SVM based on between-class relative density information (FSVM-WD). These two methods overcome the inherent defect that traditional methods are not accurate in the estimation of prior data distribution. Most of these methods set the fuzzy value of minority class instances to 1 to highlight their importance, but these do not consider the distribution characteristics of minority class instance.

In this paper, we propose a new relative density-based intuitionistic FSVM termed RIFSVM to deal with both the problem of class imbalance and noise/outlier. In our proposed model, the relative density obtained by the *K*-nearest neighbors-based probability density estimation (KNN-PDE) method [30,31] is utilized to calculate the membership degree and non-membership degree of instances. Because the relative density is adaptive for different data distribution types, and can well reflect the prior distribution information of data, it is more robust than the Euclidean distance-based measure. In addition, this method does not need to obtain the density information of all sample points, but only needs to calculate the distance between *k* neighbors of each sample and estimate its probability density distribution in feature space. Then, for instances of the majority class, we calculate the score function according to their membership degree and non-membership degree, and then set the product of the score function and the imbalance ratio as the final fuzzy value. For the instances of minority classes, the membership degree is directly set to the fuzzy value, which avoids the situation that the non-membership of minority classes is too high due to sparsity, so some points are misjudged as noise. Comprehensive experimental results on the synthetic and benchmark imbalanced datasets demonstrate the superiority of the RIFSVM to the existing and state-of-the-art class imbalance classification algorithms.

The remainder of this paper is structured as follows: Fuzzy-type support vector machines and fuzzy membership functions summarised in Section 2. Our proposed algorithm is detailedly described in Section 3. The experimental results and analysis to validate the validity of the algorithm are reported in Section 4. Finally, we conclude this paper and indicate future work in Section 5.

## 2. Fuzzy-Type Support Vector Machines and Fuzzy Membership Functions

### 2.1. Fuzzy-Type Support Vector Machines (FSVMs)

Unlike standard SVM, FSVM [18,32] assigns the corresponding membership degree to each instance according to the specified membership function. Assume that we have a binary classification problem with the dataset $T=\{\left(x_{i},y_{i}\right)|i=1,2,\cdots ,N\}$, where $x_{i}\in R^{d}$ represents an *d*-dimensional input vector, and $y_{i}\in \left\{1,-1\right\}$ refers to the corresponding class label. Then, for the given $T_{s}=\left\{\left(x_{i},y_{i},s_{i}\right)|i=1,2,\cdots ,N\right\}$, where $s_{i}$ denotes the fuzzy membership value of the *i*-th instance, which indicates the significance of the associated instance, the goal of FSVM is to learn parameters (w,b) of a hypothesis $h\left(x\right)=\langle w,\varphi (x_{i})\rangle +b$ from the optimization problem:

(1) $$\begin{array}{c} \min_{w,b}\frac{1}{2}{∥w∥}^{2}+C\sum_{i=1}^{N}s_{i}\xi_{i}\\ s.t.y_{i}\left(w^{T}\varphi \left(x_{i}\right)+b\right)+\xi_{i}\geq 1,\xi_{i}\geq 0,i=1,2,\ldots ,N, \end{array}$$

where w∈H, b∈R, tradeoff parameters C>0, $0\leq s_{i}\leq 1$ and φ is a feature mapping that maps the instance space into a high feature space. The slack variable $\xi_{i}$ denotes the measured error of the instance $x_{i}$ in FSVM, and the membership $s_{i}$ of a data point $x_{i}$ is incorporated into the objective function in the FSVM optimization problem (1), which is the only difference between the original SVM optimization problem and it.

The above optimization problem can be solved by solving the quadratic programming of the following dual form:

(2) $$\begin{array}{c} \max_{\alpha }\sum_{i=1}^{N}\alpha_{i}-\frac{1}{2}\sum_{i=1}^{N}\sum_{j=1}^{N}\alpha_{i}\alpha_{j}y_{i}y_{j}K\left(x_{i},x_{j}\right)\\ s.t.\sum_{i=1}^{N}y_{i}\alpha_{i}=0,0\leq \alpha_{i}\leq s_{i}C,i=1,\ldots N, \end{array}$$

where $\alpha =[\alpha_{1},\alpha_{2},\ldots \alpha_{N}]$ is the vector of Lagrange multipliers, and $K(x_{i},x_{j})$ being a kernel function, i.e., the inner product of the feature vectors $x_{i}$ and $x_{j}$ in the feature space $\langle \varphi \left(x_{i}\right),\varphi \left(x_{j}\right)\rangle$. It is observed that the upper bound on the value of $\alpha_{i}$ differs between the dual optimization problems for the standard SVM and FSVM.

The optimal values $\alpha^{*}$ can be obtained by solving the optimization (2), and the optimal weight vector $w^{*}$ and $b^{*}$ can be expressed as [13]:

(3) $$w^{*}=\sum_{i=1}^{N}{\alpha^{*}}_{i}y_{i}\varphi \left(x_{i}\right)$$

and

(4) $$b^{*}=y_{i}-\sum_{i=1}^{N}{\alpha^{*}}_{i}y_{i}K\left(x_{i},x_{j}\right)$$

respectively. Then the decision function can be expressed as:

(5) $$h\left(x\right)=sign\left(\sum_{i=1}^{N}{\alpha^{*}}_{i}y_{i}K\left(x_{i},x\right)+b^{*}\right).$$

The fuzzy value $s_{i}$ represents the weight of parameter $\xi_{i}$ in the objective function, reflecting the importance of the corresponding instance to the classification hyperplane. Then, the classification hyperplane can be made more reasonable by giving small fuzzy MVs for the noises and outliers. Obviously, FSVM can effectively handle outliers and noise with the help of well-defined membership functions. Therefore, defining an appropriate fuzzy membership function (MF) becomes a key issue to improve the generalization performance of FSVM.

### 2.2. Common Fuzzy MFs and Their Limitations for Class Imbalance Learning

Here, we briefly discuss some classical and new informative heuristic membership functions for general purposes, such as linear and exponential fuzzy functions [24], and the Gaussian fuzzy [33] function:

(6) $$\mu_{lin}\left(x_{i}\right)=1-\frac{d_{i}}{\max(d_{i})+\Delta };$$

(7) $$\mu_{\exp}\left(x_{i}\right)=\frac{2}{1+\exp(\beta d_{i})},\beta \in \left[0,1\right];$$

(8) $$\mu_{gau}\left(x_{i}\right)=\exp\left(-\frac{{∥d_{i}-\mu ∥}^{2}}{2\sigma^{2}}\right),$$

where $d_{i}$ indicates the Euclidean distance between the instance $x_{i}$ and the reference objects, Δ is a small positive value and β is a parameter that determines the degree of the decay, and μ and σ being the separate mean and standard deviations to be tuned during the training process.

The popular three distance reference objects contain within-class centroid, real separating hyperplane, and estimated sphere centroid. Relevant experiments in [29] show that when the datasets are approximatively subject to the standardized normal distribution, the within-class centroid reference is reliable. However, it is not a reliable reference anymore for the datasets with non-normal distribution, e.g., the datasets with small disjunctions or manifold distribution. The estimated sphere centroid reference has the same drawbacks, especially on the highly imbalanced datasets. The real separating hyperplane reference is significantly better than the two reference object. However, it is still sensitive to the degree of imbalance due to the initial separation hyperplane would be biased towards the minority class. That is, the classification model obtained by is still biased toward one of the classes.

The fuzzy value of these methods are based on the MVs of instances to their own classes, ignoring the uncertainty of instances. However, only assigning fuzzy values to instances according to the membership degree of a certain class of sample points cannot accurately describe the distribution information of instances. Therefore, we are interested in intuitionistic fuzzy sets proposed by Ha et al. [21], which considers both the membership and the non-membership degrees of instances.

### 2.3. Intuitionistic Fuzzy MF and Its Limitation for Class Imbalance Learning

**Definition** **1**. *Let X be a nonempty set, then an intuitionistic fuzzy set in a universe X, denoted A, is defined as:*

(9) $$A=\{\left(x,\mu_{A}\left(x\right),\nu_{A}\left(x\right)\right)\left|x\in X\right\},$$

*where $\mu_{A}:X\to \left[0,1\right]$ and $\nu_{A}:X\to \left[0,1\right]$ are the degrees of membership and nonmembership functions of x∈X respectively, and $0\leq \mu_{A}\left(x\right)+\nu_{A}\left(x\right)\leq 1$.*

The score function of x∈X can be determined as follows:

(10) $$H\left(x\right)=\frac{1-\nu_{A}\left(x\right)}{2-\mu_{A}\left(x\right)-\nu_{A}\left(x\right)},$$

The score function calculates the score values of instances based on their membership and non-membership values. These values can be used to compare the levels of membership among several examples of the same class.

For a binary classification problem, consider intuitionistic fuzzy sets. The instances can be converted into an intuitionistic fuzzy number (IFN) as: $T=\{\left(x_{i},y_{i},\mu_{i},\nu_{i}\right)|i=1,2,\cdots ,N\}$, where $\mu_{i}$ and $\nu_{i}$ represent the degrees of membership function and nonmembership functions of $x_{i}$ respectively. Next, the membership and nonmembership functions for each instance are defined as follows.

(1) Membership Function:

(11) $$\mu \left(x_{i}\right)=\left\{\begin{array}{c} 1-\frac{∥\varphi \left(x_{i}\right)-c_{+}∥}{R_{+}+\delta },y_{i}=+1,\\ 1-\frac{∥\varphi \left(x_{i}\right)-c_{-}∥}{R_{-}+\delta },y_{i}=-1, \end{array}\right.$$

where δ>0 is an adjustable parameter, and the $c_{+}$ ($c_{-}$) indicates the center of the positive (negative) class. The $R_{+}$ ($R_{-}$) indicates the radius of the positive (negative) class. The φ is a feature mapping that maps the instance space into some feature space. The $∥\varphi \left(x_{i}\right)-c_{\pm }∥$ represents the distance from input instance to the corresponding class center. The centers of two class can be written as:

(12) $$c_{\pm }=\frac{1}{N_{\pm }}\sum_{y_{i}=\pm 1}\varphi (x_{i}),$$

where $N_{+}$ and $N_{-}$ represent the number of positive and negative instances respectively.

The radius of two classes can be measured by

(13) $$R_{\pm }=\max_{y_{i}=\pm 1}∥\varphi \left(x_{i}\right)-c_{\pm }∥,$$

where

(14) $$\begin{array}{cc} \; & ∥\varphi \left(x_{i}\right)-c_{+}∥=\sqrt{{∥\varphi \left(x_{i}\right)-c_{+}∥}^{2}} \end{array}$$

(15) $$\begin{array}{cc} = & \sqrt{K\left(x_{i},x_{i}\right)+{\left(\frac{1}{N_{+}}\sum_{y_{i}=+1}\varphi (x_{i})\right)}^{2}-2\varphi \left(x_{i}\right)\frac{1}{N_{+}}\sum_{y_{i}=+1}\varphi (x_{i})} \end{array}$$

(16) $$\begin{array}{cc} = & \sqrt{K\left(x_{i},x_{i}\right)+\frac{1}{N_{+}^{2}}\sum_{y_{m}=+1}\sum_{y_{n}=+1}K(x_{m},x_{n})-\frac{2}{N_{+}}\sum_{y_{j}=+1}K(x_{i},x_{j})}. \end{array}$$

(2) Nonmembership Function:

The non-membership function is defined as:

(17) $$\nu \left(x_{i}\right)=\left(1-\mu \left(x_{i}\right)\right)\rho \left(x_{i}\right),$$

where $0\leq \mu \left(x_{i}\right)+\nu \left(x_{i}\right)\leq 1$, and $\rho (x_{i})$ is written as:

(18) $$\rho \left(x_{i}\right)=\frac{\left|\{x_{j}\left|∥\varphi \left(x_{i}\right)-\varphi \left(x_{j}\right)∥\leq \beta_{1},y_{j}\neq y_{i}\right\}\right|}{\left|\{x_{j}\left|∥\varphi \left(x_{i}\right)-\varphi \left(x_{j}\right)∥\leq \beta_{1}\right\}\right|},$$

where $\beta_{1}>0$ is a parameter, and the distance between two instances in the inner product space can be expressed as

(19) $$\begin{array}{c} ∥\varphi \left(x\right)-\varphi \left(x^{\prime }\right)∥=\sqrt{K\left(x,x\right)+K\left(x^{\prime },x^{\prime }\right)-2K\left(x,x^{\prime }\right)}. \end{array}$$

We define the score function for a given IFN as:

(20) $$\begin{array}{c} H\left(x_{i}\right)=\left\{\begin{array}{cc} \mu (x_{i}), & \nu (x_{i})=0,\\ 0, & \mu \left(x_{i}\right)<\nu \left(x_{i}\right),\\ \frac{1-\nu (x_{i})}{2-\mu (x_{i})-\nu (x_{i})}, & others. \end{array}\right. \end{array}$$

**Figure 1:**
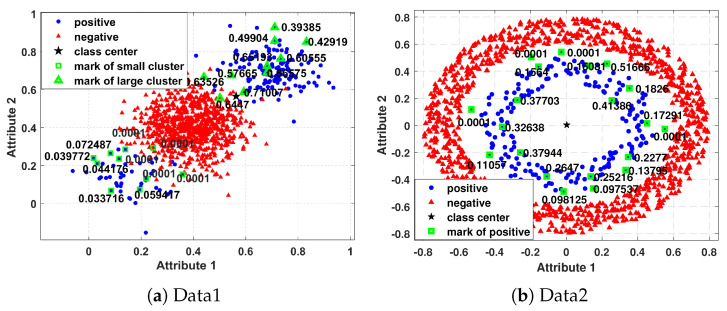
The fuzzy membership values of some positive instances of Data1 and Data2 calculated by IF.

As we know, the data distribution is generally supposed to obey a Gaussian distribution. The centroid of standardized normal distribution data always approximates the mean of the distribution. Therefore, taking the centroid as a reference can well estimate the importance of each instance. However, the distribution of data in many real-world applications is often complicated, such as the dataset of Figure 1, which follows small disjunctions or manifold distribution. This led to the within-class centroid no longer being a reliable reference, because it could neither describe the manifold structure nor catch the small subclusters well.

In Figure 1a, the instances of group *i* satisfy $x_{i}∼N\left(\mu_{i},\sigma \right)$, where $\mu_{1}=\left(0.4,0.4\right)$, $\mu_{2}=\left(0.15,0.15\right)$, $\mu_{3}=\left(0.7,0.7\right)$ and σ=[0.1,0;0,0.1]. The numbers of these three groups are 800, 50, and 150 respectively. The instances of these three groups are divided into two classes, where the instances of group 1 are set to negative class and the instances of group 2 and group 3 are set to the positive class. So we obtain an imbalanced dataset with an imbalanced ratio of 1:4. In Figure 1b, the centroid of rings is (0,0), the width of the ring equals 0.2, and the inner radius of negative and positive classes is 0.6, and 0.3 respectively, we obtain an imbalanced dataset with an imbalanced ratio of 1:5.

In Figure 1, we use IF to assign fuzzy values to some instances of Data1 and Data2. From Figure 1a, we can find that the fuzzy values of instances of small clusters of positive instances are generally low because the class center of positive instances is biased toward large clusters. Moreover, the fuzzy values of the instances that are closer to the class center in the small cluster are much smaller than that of the instances that are farther from the class center in the large cluster, and some instances in the small cluster are treated as noise. It can be seen that the fuzzy value obtained by the IF algorithm is unreasonable when the data hold small disjunctions. From Figure 1b, we can find that the fuzzy values of positive instances are generally low because the within-class centroid cannot describe the manifold structure well. Moreover, some important positive instances that are close to negative instances are judged as noise. This is because the instances are surrounded by negative instances, leading to many negative instances in the *k*-nearest neighbor, so the obtained non-membership degree will be greater than the membership degree, and such instances will be judged as noise.

As we all know, the centroid of data obeying the Gaussian distribution always approximates the mean of the distribution. Therefore, taking the centroid as a reference can well estimate the importance of each instance. However, for the data (especially imbalanced data) obeying non-Gaussian distribution, it is unreasonable to calculate the membership degree based on the class center. Meanwhile, as for the normal SVM, FSVMs are also impacted by class imbalance. Meanwhile, in most cases, to highlight the importance of the minority class, most FSVM series algorithms for class imbalance learning generally set the MVs of the minority class instances to 1, so ignore the distribution characteristics of the minority class itself.

To solve the problem discussed above, our work is based on the following two motivations:

- To provide a more reliable measure to estimate the importance of each instance.
- To propose a more preferable fuzzy MF to ensure the fairness of the classification method.

## 3. Relative Density-Based Intuitionistic Fuzzy Support Vector Machines (RIFSVM)

The relative density-based intuitionistic fuzzy membership approach is proposed first. Then, the relative density-based intuitionistic fuzzy SVM is proposed by using the density-based intuitionistic fuzzy membership for class imbalance learning.

### 3.1. Relative Density Estimate Based on a k-Nearest-Neighbor Distances

Based on the above analysis, it can be seen that, for some imbalanced datasets with special distribution, it is unreasonable to calculate the degree of membership and non-membership of the instance based on the within-class centroid criterion and calculate the fuzzy value of the instance according to the intuitionistic fuzzy value as well. Therefore, we should take advantage of the prior information of the instances, instead of giving the fuzzy values to instances by assuming that the instance conform to a certain distribution.

It is obvious that it will be easier to discriminate between regular instances and outliers/noise if we can accurately estimate the probability density of each case. However, it is incredibly challenging to quantify the probability density accurately in high dimensional feature space. Instead of precisely measuring the probability density of each instance, previous works [29] have proposed an alternative scheme in which we can determine the proportional relation of the probability densities between any two instances. The information that reflects the proportional relation is called relative density [34]. Here, we estimated the relative density using the K-nearest neighbors-based probability density estimation (KNN-PDE) [30,31] method. The KNN-PDE estimates the probability density distribution of the instances in the multi-dimensional continuous space according to the K-nearest neighbor distance of each instance. The KNN-PDE result can approximately converge to the real probability density distribution when the number of instances reaches infinity [35].

Suppose a dataset includes $N_{+}$ positive instances and $N_{-}$ negative instances, and $N_{+}+N_{-}=N$. In this paper, the positive class stands for the minority class and the negative class stands for the majority class. Then for each instance, we find its *K*-th nearest neighbor and denote the distance between them as $d_{i}^{k}$. It is not difficult to see that the larger $d_{i}^{k}$, the sparser the distribution of instance, that is, the smaller the density. As we know, noise and outliers are often in the low-density region, thereby we can estimate the importance of each instance by $d_{i}^{k}$. We define $1/d_{i}^{k}$ as the relative density. Obviously, it assigns greater values to instances of high density and lower values to instances of low density, such as noise and outliers. We can also obtain the proportionality of the relative density of any two instances as follows:

(21) $$\frac{1/d_{i}^{k}}{1/d_{j}^{k}}=\frac{d_{j}^{k}}{d_{i}^{k}}.$$

This proportional relation equals the inverse of the ratio of the k-nearest neighbor distances between these two instances. Choosing an appropriate parameter *k* may be quite important for the relative density. If the *k* is too large, some normal instances may be misjudged as noise. If the *k* is too small, some noise and outliers would not be identified. In this paper, we set $k=\sqrt{N}$ by experience, where *N* is the number of instances.

Next, we introduce the two relative densities: within-class relative density and between-class relative density.

(1) The *within-class relative density* refers to the relative density of an instance in its own class. For example, the within-class relative density of positive instance $x_{i}$ is $1/d_{i}^{k^{+}}$, the $d_{i}^{k^{+}}$ is the distance between $x_{i}$ and the $k^{+}$-th nearest neighbor in the positive instances, where $k^{+}=\sqrt{N_{+}}$. The larger the value of $d_{i}^{k^{+}}$, the lower the relative density of the instance, then the lower the probability that the instance belongs to this class.
(2) The *between-class relative density* refers to the relative density of an instance in another class. The between-class relative density of positive instance $x_{i}$, for instance, is $1/d_{i}^{k^{-}}$, the $d_{i}^{k^{-}}$ is the distance between $x_{i}$ and the $k^{-}$-th nearest neighbor in the negative instances, where $k^{-}=\sqrt{N_{-}}$. The larger the value of $d_{i}^{k^{-}}$, the lower the relative density of the instance, then the farther $x_{i}^{+}$ is from the negative instances, that is the lower the probability that the instance belongs to the negative class.

### 3.2. Relative Density-Based Intuitionistic MFs for Class Imbalance Learning

Based on the fact above, we proposed a combined formulation of MFs based on relative density-based intuitionistic, which provides the membership values for instances to satisfy two goals:

- to lessen the impact of class imbalance;
- to reduce the negative impact of noise and outliers.

(1) Determination of fuzzy value of majority class

For the instance of the majority class, the MV is calculated by the within-class relative density. Here, the exponential function is used to give the MVs of the instances. Instances with a larger $d_{i}^{k^{-}}$ have a smaller relative density, so they should be given lower MVs. The specific calculation process is given as follows.

First, we calculate the MVs of instances according to the following membership degree function:

(22) $$\mu^{-}\left(x_{i}\right)=\frac{2}{1+\exp\left(\frac{d_{i}^{k^{-}}}{\max\{d_{1}^{k-},d_{2}^{k^{-}}\cdots ,d_{N^{-}}^{k^{-}}\}}\right)}.$$

Then, we calculate the non-membership values of instances according to the following non-membership degree function:

(23) $$\nu^{-}\left(x_{i}\right)=\left(1-\mu^{-}\left(x_{i}\right)\right)\rho \left(x_{i}\right),$$

where the $\rho (x_{i})$ is defined as:

(24) $$\rho \left(x_{i}\right)=\frac{2}{1+\exp\left(\frac{d_{i}^{k^{+}}}{\max\{d_{1}^{k^{+}},d_{2}^{k^{+}}\cdots ,d_{N^{+}}^{k^{+}}\}}\right)}.$$

Note that the $\rho (x_{i})$ is calculated by the between-class relative density of instance.

Then, according to the MVs and non-MVs of the instances, we define the score function as:

(25) $$H\left(x_{i}\right)=\left\{\begin{array}{c} 0,\mu^{-}\left(x_{i}\right)<\nu^{-}\left(x_{i}\right),\\ \frac{1-\nu^{-}\left(x_{i}\right)}{2-\mu^{-}\left(x_{i}\right)-\nu^{-}\left(x_{i}\right)},others. \end{array}\right.$$

It can easily distinguish normal instance from noises and outliers [21]. Finally, to lessen the influence of class imbalance, the fuzzy values of negative instances can be defined as:

(26) $$s_{i}=IR\cdot H\left(x_{i}\right),$$

where IR is the minority-to-majority class ratio.

(2) Determination of fuzzy value of minority class

As is well known, there are few instances in the minority class. If the above method is used to calculate the fuzzy value, a large non-membership degree will be obtained, which will make the instance misjudged as noise. It can be seen that it is unreasonable to calculate the fuzzy value of the minority class using the majority class’s fuzzy value. It is also unreasonable to set the fuzzy value of the minority class to 1 directly like FSVM-CIL series algorithms.

Therefore, we directly set the membership degree of minority class instances as the fuzzy value. In this way, we not only give high fuzzy values to minority class instances but also fully utilize the prior information of instances.

(27) $$\mu^{+}\left(x_{i}\right)=\frac{2}{1+\exp\left(\frac{d_{i}^{k^{+}}}{\max\{d_{1}^{k^{+}},d_{2}^{k^{+}}\cdots ,d_{N^{+}}^{k^{+}}\}}\right)},$$

(28) $$s_{i}=\mu^{+}\left(x_{i}\right).$$

Since the fuzzy values of both majority and minoriy instances are calculated based on relative density, the above algorithm for calculating fuzzy values is denoted as RIF.

As we can see, the fuzzy values of the majority class instances are designed by incorporating the relative density measure into the intuitionistic fuzzy numbers. In this way, with the help of the strong capture ability of the relative density to prior information and the strong recognition ability of the intuitionistic fuzzy score function to outliers and noises, the fuzzy function can be adopted to identify possible noises and outliers existing in the majority class. Additionally, the participation of imbalance ratio reduces the effect of class imbalance. For the minority instances, to lessen disadvantage of being misjudged as noise points because the instances are too sparse, the fuzzy values of minority class instances is directly assigned as the MVs calculated by relative density without the non-MVS.

**Figure 2:**
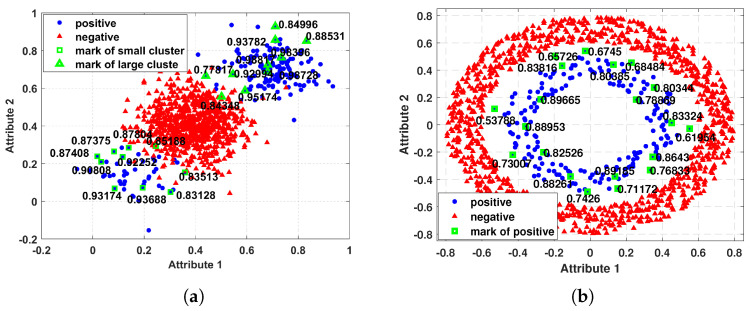
Fuzzy membership values of some positive (minority) class instances calculated by RIF. (**a**) Fuzzy membership values of Data1; (**b**) Fuzzy membership values of Data1.

In order to explore the rationality of RIF, we compute the fuzzy value of Data1 and Data2 using the proposed RIF method and mark the fuzzy values of 20 instances which are the same as those in Figure 1, and the results are displayed in Figure 2. In Figure 2a, it is obvious that the fuzzy values of the small clusters instances are not too small as shown in Figure 1a, so they are not misjudged as noise. Instead, it follows the distribution characteristics of instances, assigning smaller fuzzy values to sparsely distributed points and larger fuzzy values to densely distributed sample points. Instead, it follows the distribution characteristics of instances, assigning smaller fuzzy values to sparsely distributed points and larger fuzzy values to densely distributed instances. From the Figure 2b, we can find that the instance on the edge is not misjudged as noise, and given a reasonable fuzzy value. It can be seen that for such imbalanced datasets with non-normal distribution, our RIF method is relatively reasonable.

### 3.3. Relative Density-Based Intuitionistic Fuzzy Support Vector Machines

By integrating the relative density-based intuitionistic MVs into FSVM, we propose a novel relative density-based intuitionistic fuzzy support vector machine for imbalanced learning with noise and outliers. The corresponding optimization problem can be expressed as:

(29) $$\begin{array}{c} \min_{w,b}\frac{1}{2}{∥w∥}^{2}+C\sum_{i=1}^{N}s_{i}\xi_{i}\\ s.t.y_{i}\left(w^{T}\varphi \left(x_{i}\right)+b\right)+\xi_{i}\geq 1,\xi_{i}\geq 0,i=1,2,\ldots ,N, \end{array}$$

where $s_{i}=\left\{\begin{array}{c} \mu^{+}\left(x_{i}\right),y_{i}=1,\\ IR\cdot H\left(x_{i}\right),y_{i}=-1, \end{array}\right.$ and the $\mu^{-}\left(x_{i}\right)$, $\nu^{-}\left(x_{i}\right)$, $\mu^{+}\left(x_{i}\right)$, and $H(x_{i})$ are shown in (22), (23), (27) and (25) respectively.

At the same time, when $C^{-1}/C^{+1}=IR$ and $C^{+}=C$, we can also write the objective function of the above optimization problem in the following cost-sensitive form:

(30) $$f\left(w,b,\xi \right)=\frac{1}{2}{∥w∥}^{2}+C^{+}\sum_{y_{i}=+1}s_{i}\xi_{i}+C^{-}\sum_{y_{i}=-1}s_{i}\xi_{i},$$

where $s_{i}=\left\{\begin{array}{c} \mu^{+}\left(x_{i}\right),y_{i}=1,\\ H\left(x_{i}\right),y_{i}=-1. \end{array}\right.$

To solve the optimization problem of RIFSVM in (29), the dual form of it can be written as:

(31) $$\begin{array}{cc} \max_{\alpha } & \sum_{i=1}^{N}\alpha_{i}-\frac{1}{2}\sum_{i=1}^{N}\sum_{j=1}^{N}\alpha_{i}\alpha_{j}y_{i}y_{j}K\left(x_{i},x_{j}\right)\\ s.t. & 0\leq \alpha_{i}^{}\leq s_{i}C,\sum_{i=1}^{N}y_{i}\alpha_{i}=0,i=1,2,\ldots ,N \end{array}$$

where $\alpha ={\left[\alpha_{1},\ldots ,\alpha_{N}\right]}^{T}$ is the Lagrange multiplier. $K\left(x_{i},x_{j}\right)=\varphi {\left(x_{i}\right)}^{T}\varphi \left(x_{j}\right)$ is the kernel function satisfying Mercer’s theorem.

The optimal values $\alpha^{*}$ can be obtained by solving the optimization (31), and the decision function of a RIFSVM can be expressed as:

(32) $$h\left(x\right)=sign\left(\sum_{i=1}^{N}{\alpha^{*}}_{i}y_{i}K\left(x_{i},x\right)+b^{*}\right),$$

where $w^{*}$ and $b^{*}$ are shown in (3) and (4).

The above training process is summarized as Algorithm 1.
**Algorithm 1** RIFSVM algorithm**Input:** Training dataset $T=\{\left(x_{1},y_{1}\right)\cdots \left(x_{N},y_{N}\right)\}$, Penalty parameter *C*, kernel spread parameters h>0.**Output:** The decision function (5). 1:The training sample *T* is divided into $T_{+}$ and $T_{-}$, $T_{+}$ is the set of minority instances, and $T_{-}$ is majority instances. Calculate the numbers of minority and majority instances and denote them as $N_{+}$ and $N_{-}$ respectively; 2:Calculate the nearest neighbor parameter $k^{+}=\sqrt{N_{+}}$, $k^{-}=\sqrt{N_{-}}$; 3:For the instance $x_{i}\in T_{-}$, calculate $d^{k^{-}}$ and calculate the membership degree $\mu^{-}\left(x_{i}\right)$ of the majority class instance according to (22); 4:For the instance $x_{i}\in T_{-}$, calculate $d^{k^{+}}$ in the minority class and calculate the nonmembership degree $v^{-}\left(x_{i}\right)$ of the instance according to (23); 5:According to the obtained $\mu^{-}\left(x_{i}\right)$ and $\nu^{-}\left(x_{i}\right)$ and (25), the final fuzzy value $s(x_{i})$ of the majority instances is calculated by using according to (26); 6:For the instance $x_{i}\in T_{+}$, calculate $d^{k^{+}}$ and calculate the fuzzy value $s(x_{i})$ of the minority instance according to (28); 7:Train NIFSVM model (29) to obtain the decision function.

## 4. Experiments and Analysis

In this section, we explore the performance and superiority of our proposed algorithms using synthetic and benchmark datasets. The five-fold cross-validation technique [36] is used in this paper to choose all of the parameters for these algorithms. The Gaussian kernel function $K\left(x_{1},x_{2}\right)=\exp\left(-h{∥x_{1}-x_{2}∥}^{2}\right)$ is used for all data sets, kernel spread parameters *h* are roughly chosen within $\left\{2^{-5},2^{-4}\cdots ,2^{4},2^{5}\right\}$. The tradeoff parameters *C* of our model are selected from the set of $\{10^{-5},10^{-4},\cdots ,10^{4},10^{5}\}$. All the experiments are carried out on a desktop PC with Intel(R) Xeon(R) CPU (3.30 GHz) and 32 GB RAM under the MATLAB 2019a programming environment.

### 4.1. Evaluation Metrics for Imbalanced Classification

The accuracy-based evaluation metric is usually used to assess the performance of the general classification method. However, it is no longer an appropriate metric for the imbalanced classification method since the effect of minority class on accuracy is smaller than that of majority class. In this paper, we use G-Mean, F-Measure, and AUC to evaluate the performance of imbalanced dataset classification. The G-Mean, a comprehensive measure of minority and majority class, denotes the geometric mean of sensitivity and specificity [37]. A reasonably high value of both True Positive and True Negative ensures a high G-Mean value. The F-Measure denotes the harmonic mean of Precision and Recall A high value of F-Measure means that both sensitivity and Precision are high simultaneously, and the AUC (the area under receiving operator characteristic curve) is another evaluation measure of classification performance in imbalanced problems because the area under the ROC graph is not sensitive to the distribution of two classes.

### 4.2. Experiments on the Synthetic Imbalanced Datasets

**Figure 3:**
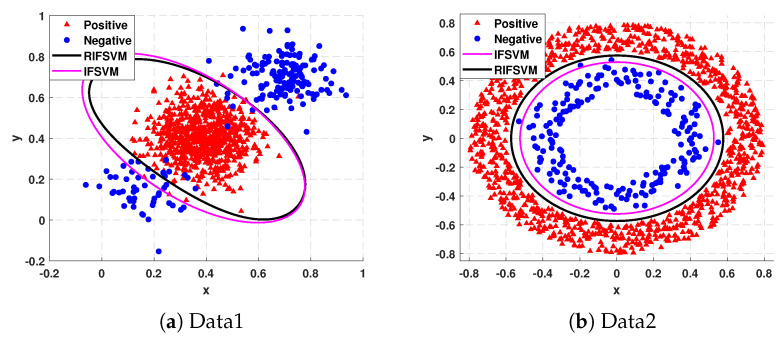
Plots for comparing the classification boundaries of IFSVM and RIFSVM on the Data1 of Figure 1.

To intuitively show the effectiveness of the proposed model in dealing with class imbalance learning, we classify the two synthetic datasets in Figure 1 using IFSVM and RIFSVM, respectively. The experimental results are shown in Figure 3. In addition, for these two datasets, we trained the model on 80% of the instances and tested the model on the remaining 20%. The experiments are performed 10 times, and Table 1 lists the average G-Means, F-Measures, and AUC values. The best results are denoted in boldface.

From Figure 3a and Table 1, we can find that for Data1, the IFSVM has a higher misclassification rate for small cluster instances of minority instances. This is because we can see from Figure 1a that the instances of the small cluster are endowed with lower final fuzzy values, especially some instances of the small cluster are judged as noise, resulting in these important instances will no longer contribute to separating the hyperplane and are likely to be misclassified. However, the classification result obtained by RIFSVM is much better, and the misclassification rate for the instances of the small cluster is also lower. From Figure 3b and Table 1, we can find that the misclassification rate of boundary instances of minority class by IFSVM is higher than that by RIFSVM. This is because the non-membership degree of these instances calculated by IFSVM is higher, which leads to the smaller fuzzy value, and then these instances are misclassified. Similarly, the evaluation measure G-mean, F-Measure, and AUC of RIFSVM are all higher than those of IFSVM. It can be concluded that our method is effective when tackling imbalance datasets.

### 4.3. Experiments on Benchmark Datasets

In this section, we selected twenty datasets from the keel and UCI repositories in order to assess the performance of the proposed RIFSVM in tackling class imbalance classification. A complete description of each dataset is presented in Table 2. Some multi-classification problems were converted into binary classification problems by a one-versus-others strategy. In addition, we obtain some imbalanced datasets from the Yeast and Block datasets using different class combinations in order to obtain datasets with various imbalance ratios. The positive class and negative class columns in Table 2 contain a complete list of the class combinations for these generated datasets. The IR varies from 1:1.38 to 1:42.72 and the the number of attributes varies from 3 to 90. All attributes are normalized into the interval [0,1].

#### 4.3.1. Experimental Procedure and Results

We performed experiments on all the datasets in Table 2 to further confirm the effectiveness of the proposed method and compare it to IFSVM and other imbalance learning approaches.

(1) IFSVM [20]: It uses the membership and non-membership calculated by the sample distribution information to determine the fuzzy value of the instance. The parameter $\delta =10^{-4}$, and $\beta_{1}=\min\left(r\right)/5$, where $r=(\max\left(R_{n}\right),\max\left(R_{p}\right))$.
(2) ACFSVM [26]: It assigns the fuzzy value to the majority class instances based on the affinity and class probability and assigns 1 as the fuzzy value of minority class instances to highlight the importance of minority instances. The kernel nearest neighbor parameter *k* is chosen from the set {3,5,7,9,11} and the parameter ρ is selected from the set {0.1,0.5,1,2,3,5,7,10,20}.
(3) GFSVM [25]: It is the supplement and extension of FSVM-CIL, a new distance and a new fuzzy value function, namely Gaussian fuzzy function, is proposed. The parameters μ and σ in the Gaussian function are selected from sets {0.01,0.05,0.1,0.2,0.3,0.4,0.5,0.7} and $\left\{2^{-6},2^{-5},\ldots ,2^{5},2^{6}\right\}$, respectively.
(4) EFTWSVM [27]: It uses the information entropy of instances to calculate fuzzy values to minority instances, it fully utilizes the prior information of instances; it assigns 1 as the fuzzy value of minority class instances. Then the fuzzy values obtained are used in the improved TWSVM. The parameters β of EFTWSVM is taken as 0.05, the value of *K* for K-NN is 10.
(5) RFLSTSVM [28]: It assigns a more robust fuzzy value to majority class instances and assigns 1 as the fuzzy value of minority class instances, then trains the LSTSVM model on this training set. The parameter $k_{0}$ of RFLSTSVM is selected from the set {0.5,1,1.5,2,2.5}.
(6) FSVM-WD and FSVM-BD [29]: They are all proposed based on relative density, and the sum of fuzzy values of positive and negative class instances is set as 1 to ensure the robustness of the model. The distinction is that while FSVM-WD is based on information about within-class relative densities, FSVM-BD is based on the between-class relative densities. These two methods use the same strategy to calculate the fuzzy values of instances. The parameters of them are set as λ=0.01/N, $k=\sqrt{N}$.

Note that several different algorithms are listed above. They adopt different strategies when calculating the fuzzy values of minority and majority classes. In addition, for some algorithms that set the fuzzy value of minority classes directly to 1, their classification models are also different. The effectiveness of the RIFSVM method is explored by comparing it with different fuzzy value setting methods and different classification model algorithms.

The experiment consisted of two parts. In the first part, the accuracy of minority and majority classes (Se and Sp) of eight algorithms on some datasets are compared. Then, the classification performances of eight algorithms on 20 datasets are compared. All the parameters are chosen by five-fold cross-validation based on G-Means.

**Figure 4:**
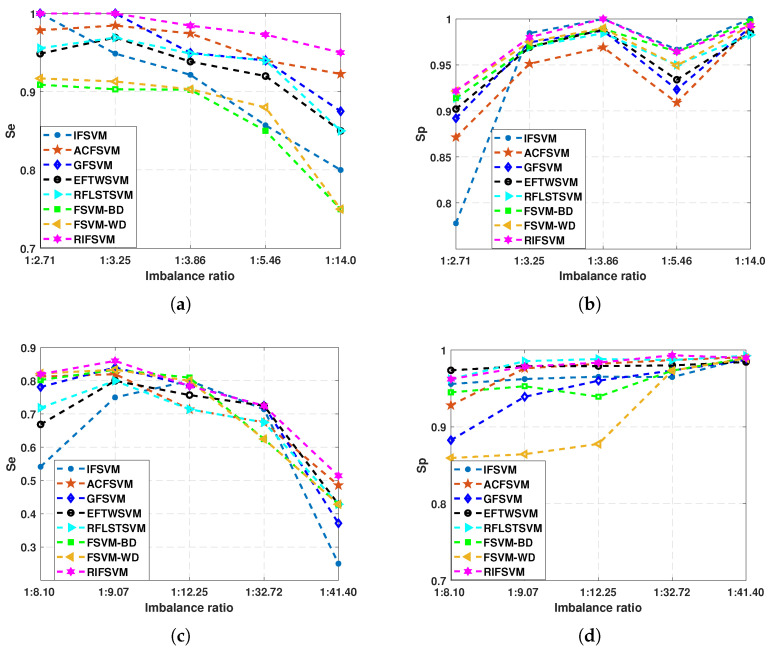
(**a**,**b**) are the accuracies of minority and majority class of the eight algorithms on five datasets with different imbalance ratios. (**c**,**d**) are the accuracies of minority and majority class of the eight algorithms on the Yeast dataset with different imbalance ratios.

First, to compare the accuracies of eight algorithms on positive and negative classes, we performed the experiments on five datasets in Table 2, which contain Wine, Vehicle, Abalone, Ecoli, and Libra. Their imbalance ratios are 1:2.71, 1:3.25, 1:3.86, 1:5.46, and 1:14, respectively. Except for the last Libra dataset, the IR of the other datasets have less difference. The accuracy of positive and negative classes for the seven algorithms on these five datasets is calculated, and the accuracy line chart is drawn in Figure 4a,b. In addition, Yeast1-Yeast5 are selected to explore the relationship between accuracy and imbalance ratio, the difference in imbalance ratio is significantly larger than in the previous five datasets. Their imbalance ratios are 1:8.10, 1:9.07, 1:12.25, 1:32.72, and 1:41.40, respectively. The corresponding experimental results are plotted in Figure 4c,d.

As shown in Figure 4a, the accuracy of these eight algorithms on the minority classes gradually decreases as the imbalance ratio rises, when the imbalance ratio is 1:14, the accuracy of minority classes of the seven algorithms decreases significantly. The accuracy of the proposed RIFSVM is slightly higher than that of other algorithms on the minority classes. When the imbalance ratio becomes large, the accuracy of RIFSVM decreases less than that of other algorithms. The results in Figure 4b show that the accuracy of these seven algorithms on majority classes gradually increases as the imbalance ratio increases. When the imbalance ratio is 1:5.46, the accuracy of majority classes of the seven algorithms is relatively low, which may be related to the dataset. The RIFSVM algorithm still outperforms other algorithms in the accuracy of most classes. From the results shown in Figure 4c, it can be seen that the accuracy of seven algorithms on the minority classes showed a downward trend as the imbalance ratio increased. When the imbalance ratio is 1:9.07, the accuracy of the eight algorithms is relatively high, which may be data related. The accuracy of the our algorithm is slightly better than the other seven algorithms for the minority classes because the algorithm uses relative density to calculate the fuzzy value of the instances and makes full use of the distribution characteristics of the instances. From the results of Figure 4d, we can find that the accuracy of these seven algorithms on majority class increases slowly as the imbalance ratio increases. When the imbalance ratio reaches the maximum, the accuracy of the eight algorithms reaches the highest, indicating that when the imbalance is relatively large, the classifier will still bias toward the majority class. In addition, it can be seen that the accuracy of the FSVM-WD algorithm on the majority class is lower than that of other algorithms. It may be because the algorithm only uses the within-class relative density of instances, which results in inaccurate fuzzy values.

In the second part, we conducted experiments on each of datasets provided in Table 1 in comparison to the other six imbalanced classification algorithms above. Experiments are repeated 10 times, and the G-Means, F-Measures, and AUC are recorded in Table 3. The best values are highlighted in bold.

As shown in Table 3, ACFSVM achieve higher G-Mean and AUC values but obtains lower F-Mean. In fact, a high F-Measure value means high classification accuracy of minority classes. It is evident that the performance of the ACFSVM for minority classification is not high. This may be because the separation hyperplane obtained by the ACFSVM algorithm is still skewed to minority classes, which contributes to the low classification accuracy of minority classes. The performance of the GFSVM algorithm on all datasets is mediocre, but its F-Mean is mostly higher than the ACFSVM. This may be because GFSVM uses the same fuzzy function to calculate the fuzzy value of minority class and majority class, and the difference between majority class and majority class only depends on the imbalance ratio to constrain, which does not result in a significant deviation of the separation hyperplane, so the classification effect will not be greatly improved. EFTWSVM and RFLSTSVM algorithms form a separate hyperplane in each class during training, and their classification performance may be general since the classification accuracy of EFTWSVM and RFLSTSVM is inferior to SVM. The FSVM-BD and FSVM-WD have poor performance in G-Mean and AUC metrics, but their F-Mean is very high. It indicates that the relative density can well reflect the distribution characteristics of minority classes so that the separation hyperplane is no longer biased toward the majority class. In addition, we also find that the classification performance of FSVM-BD, FSVM-WD, and RIFSVM on the Balance dataset is significantly better than that of other methods because these three algorithms all use the relative density of instances when calculating the fuzzy value. It indicates that the relative density can well describe the distribution characteristics of the Balance dataset. On most data sets, the classification performance of the FSVM-BD and FSVM-WD is not good, which may be because the two algorithms use the normalization method to make the sum of the fuzzy values of minority and majority categories equal when calculating the fuzzy values. When the number of instances is large, the fuzzy value of the instances will become small, which leads to poor classification performance.

Finally, the proposed RIFSVM performs well in the three performance indexes of classification on most datasets. This is because RIFSVM not only uses the relative density to give the distribution information of minority and majority class instances but also combines the intuitionistic fuzzy set to give different fuzzy values of positive class sample instances. Thus, the separation hyperplane is no longer skewed to the minority class.

#### 4.3.2. Statistical Comparisons by Friedman Test

The Friedman tests [38] are used to provide a statistical comparison of the proposed RIFSVM with the existing class imbalance learning algorithms. First, we determined average ranks for the G-Mean, F-Measure, and AUC metrics of the seven methods across all datasets. For these datasets, we set the average rank of the algorithm with the best classification performance as 1 while the worst classification performance as 8.

**Figure 5:**
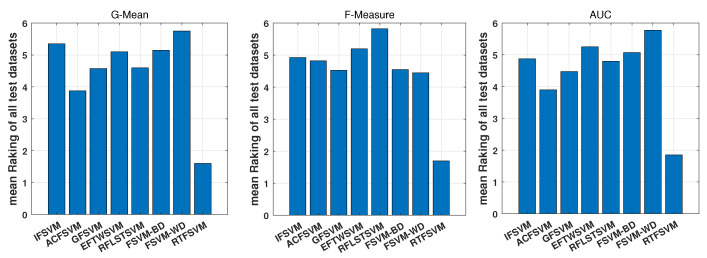
Mean ranking of all compared imbalanced classification algorithms on test datasets.

From the results in Figure 5, we can find that the proposed RIFSVM ranks first with an average score of 1.60, 1.70, and 1.85 in terms of G-Mean, F-Measure, and AUC, respectively. It demonstrates that in the mean ranking of all evaluation measures, our proposed RIFSVM performs better than other methods.

Then, the Friedman test is used to judge whether these algorithms all perform equally. Obviously, *k*(=8) compared algorithms and *n*(=20) imbalance datasets are considered in this experiments. The $r_{i}$ is the average rank of the *j*-th algorithms. Under the null hypothesis, which states that all the algorithms are equivalent, and thus their ranks should be equal, the Friedman statistic

(33) $$\begin{array}{c} \Gamma_{\chi^{2}}=\frac{12N}{k(k+1)}\left(\sum_{i=1}^{k}r_{i}^{2}-\frac{k{\left(k+1\right)}^{2}}{4}\right) \end{array}$$

is distributed according to $\chi^{2}$ with (k−1) degrees of freedom, when *n* and *k* are reasonably large. The Friedman $\tau_{\chi^{2}}$ presents a pessimistic behavior, thus the statistic

(34) $$\begin{array}{c} \tau_{F}=\frac{\left(n-1\right)\Gamma_{\chi^{2}}}{n\left(k-1\right)-\Gamma_{\chi^{2}}} \end{array}$$

is usually used, which is distributed according to the *F*-distribution with (k−1) and (k−1)(n−1) degrees of freedom. According to (34), the Friedman statistic $\tau_{F}$ of G-Mean, F-Measure, and AUC are 7.50, 6.24, and 6.04 respectively, which at significance level α=0.05 rejects the null hypothesis of equal performance.

The Nemenyi test is then employed to further identify these eight algorithms. The supposition that “the two algorithms have the same performance” is rejected with corresponding confidence if the difference between the average ranks of the two algorithms exceeds the critical difference (CD). The critical range of average ranks difference $CD=q_{\alpha }\sqrt{\frac{k(k+1)}{6n}}$ is calculated as 2.3478, where critical values $q_{\alpha }$ are based on the studentized range statistic.

**Figure 6:**
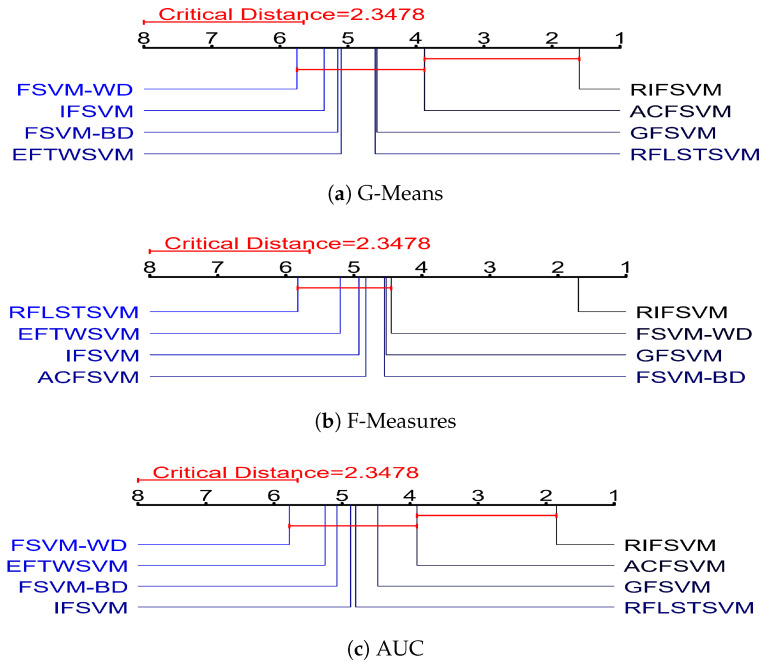
CD diagrams of the eight comparison models on the twelve benchmark datasets with three performance indexes of classification. It is clear that RIFSVM achieves statistically superior performance on the datasets with different performance indexes of classification.

The average rank of each comparing algorithms is indicated along the axis in Figure 6’s CD diagrams of G-Mean, F-Measure, and AUC on the twenty benchmark datasets. The axis has been rotated to place the highest ranks to the right. A red line connects groups of approaches that, according to the Nemenyi test, are not significantly different from one another. In each subfigure, the critical difference is also displayed above the axis.

As can be seen from Figure 6b, our algorithm is optimal in F-Meansure, and it is significantly superior and different from other algorithms. From Figure 6a,c, we can see that our algorithm is optimal in G-Mean and AUC, and is significantly superior different from other algorithms except for ACFSVM. The ACFSVM is only inferior to our method while superior to the other six algorithms in terms of G-Mean and AUC, but it’s not doing very well on the F-Measure. The FSVM-WD and FSVM-BD perform better on F-Measure but are the worst on both G-Mean and AUC. The GFSVM performs generally well in all indicators, and IFSVM, RFLSTSVM and EFTWSVM are mediocre in terms of G-Mean, F-Measure, and AUC. In general, our RIFSVM achieves statistically superior performance on the whole twenty datasets.

#### 4.3.3. Influences of Parameter k on the Performance

**Figure 7:**
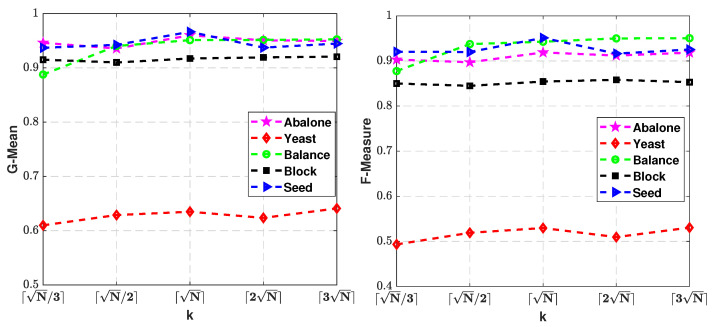
Plots of G-Means and F-Measures obtained by RIFSVM with respect to different *k* of the model on Abalone, Yeast, Balance, Block, and Seed datasets.

In order to evaluate the influence of the parameter *k* for relative density calculation on the classification performance of our proposed approach. It is selected from the set $\left\{\left\lceil \sqrt{N}/3\right\rceil ,\left\lceil \sqrt{N}/2\right\rceil ,\left\lceil \sqrt{N}\right\rceil ,\left\lceil 2\sqrt{N}\right\rceil ,\left\lceil 3\sqrt{N}\right\rceil \right\}$. The variation of the G-Means and F-measures with the variation of parameter *k* on the five chosen datasets is then plotted in Figure 7.

From the results, it can be seen that although there are some fluctuations, the performance increases at the initial stage and decreases after reaching the peak with the increase of *k*. That is, the performance of the proposed algorithms will deteriorate if *k* is too high or too low. In fact, if the *k* is set at a value that is too low, outliers and noise points might be assigned oversize weights. While the *k* is set at a value that is too high, the instances in the same category might be assigned undersized weights. The results in Figure 7 provide some reference, i.e., the performance of our method could be assured when *k* is between $\left\lceil \sqrt{N}/2\right\rceil$ and $\left\lceil 2\sqrt{N}\right\rceil$. This shows that setting the parameter *k* as $\left\lceil \sqrt{N}\right\rceil$ empirically in our experiment is reasonable. In practical applications, we recommend that the user choose the appropriate parameter *k* by themselves.

## 5. Conclusions

In this paper, the FSVM technique for class imbalance learning has been discussed. First, the shortcomings of the settings of existing intuitionistic fuzzy value on some imbalanced datasets with specific distribution and limitations of traditional FSVM-CIL series algorithms in dealing with imbalanced classification task has been analyzed. Then, the importance of digging into the prior information of the instances and merging them into the classification model has been emphasized. Inspired by the work above, a novel relative density-based Intuitionistic FSVM (RIFSVM) has been presented for imbalanced learning with outliers and noise. Specifically, the membership degree and non-membership degree of instances are calculated based on relative density and exponential decay function. According to the above membership degree and non-membership degree, the fuzzy value of majority instances is calculated by the specific score function, and the fuzzy value of minority instances is set as the membership degree. With the help of the strong capture ability of the relative density to prior information and the strong recognition ability of the intuitionistic fuzzy score function to outliers and noises, the proposed RIFSVM not only suppresses the influence of class imbalance but also reduces the impact of noises and outliers. Finally, we demonstrate the effectiveness and superiority of our proposed algorithm on synthetic and benchmark imbalanced datasets. The experimental results on the synthetic datasets show that the proposed RIFSVM can achieve a better classification boundary than IFSVM and be flexible for various types of data distribution. The experimental results on benchmark datasets demonstrate that the proposed algorithm achieves better performance than the other state-of-the-art class imbalance learning algorithms in terms of G-Means, F-Measures, and AUC with good robustness, and a statistical test is applied to verify the performance significance of the proposed method. In addition, after the influences of parameters *k* on the performance were discussed in this study, we found that when *k* is between $\left\lceil \sqrt{N}/2\right\rceil$ and $\left\lceil 2\sqrt{N}\right\rceil$, the performance of our algorithm could be guaranteed.

In the future, it will be interesting to translate the proposed approach into an efficient approach to multiple classes of problems. In addition, how to combine the relative density with other score functions to obtain reasonable fuzzy values for some other specific classification problems would be investigated in the future, too.

## Figures and Tables

**Table 1:** Classification results of IFSVM and RIFSVM on two synthetic datasets in Figure 1, the best values are in bold.

	Results (%)	Se	Sp	G-M	F-M	AUC
Data 1	IFSVM	86.50	99.00	92.75	90.81	92.75
	RIFSVM	**92.50**	99.13	95.75	94.39	95.81
Data 2	IFSVM	98.00	100.00	98.99	98.99	99.00
	RIFSVM	100.00	100.00	100.00	100.00	100.00

**Table 2:** Details of the imbalanced datasets.

Dataset	Feature	Instance	Positive	Negative	Minority Class	Majoritt Class	IR
Liver	6	345	145	200	class 1	class 2	1:1.38
Seed	7	210	70	140	class 1	others	1:2.00
Wine	13	178	48	130	class 1	others	1:2.71
Haberman	3	306	81	225	class 2	class 1	1:2.78
Glass	4	150	50	100	class 0,1,2,3	class 4,5,6	1:3.19
Vehicle	18	846	199	647	‘van’	others	1:3.25
Abalone	8	326	67	259	class 16	class 6	1:3.86
Ecoli	7	336	52	284	‘pp’	others	1:5.46
Balance	4	625	49	576	‘B’	others	1:11.76
Libra	90	360	24	336	class 15	others	1:14.00
Yeast	8	1484	429	1055	class 2	others	1:2.46
Yeast1	8	1484	163	1321	class 4	others	1:8.10
Yeast2	8	514	51	463	class 5	class 1	1:9.07
Yeast3	8	464	35	429	class 7	class 2	1:12.25
Yeast4	8	1484	44	1440	class 6	others	1:32.72
Yeast5	8	1484	35	1449	class 7	others	1:41.40
Block	10	5473	560	4913	others	class 1	1:8.77
Block1	10	5473	329	5144	class 2	others	1:9.07
Block2	10	5144	231	4913	class 3,4,5	class 1	1:21.27
Block3	10	5028	115	4913	class 5	class 1	1:42.72

Note: The feature and instance indicate the number of features and instances respectively, and IR is the imbalance ratio.

**Table 3:** Classification results obtained by eight algorithms on twenty datasets, the best values are in bold.

Dataset	Results	IF SVM	ACF SVM	GF SVM	ETW SVM	RFLST SVM	FSVM -BD	FSVM -WD	RIF SVM
Liver	Gm	67.82	60.10	64.48	66.52	65.85	67.47	64.33	69.48
	F	62.08	67.30	58.00	61.55	60.41	61.54	58.18	64.00
	AUC	69.90	66.21	65.96	68.47	68.88	68.84	65.09	**71.34**
Seed	Gm	89.21	93.62	92.44	93.65	93.33	87.04	91.05	94.63
	F	85.71	91.20	89.40	89.42	89.53	84.62	88.89	92.86
	AUC	89.29	93.89	92.50	93.39	93.75	87.50	91.01	94.64
Wine	Gm	88.19	96.17	93.24	91.35	88.19	94.28	87.71	98.06
	F	87.50	93.55	91.58	88.50	87.50	94.12	75.00	94.71
	AUC	88.89	96.24	93.80	91.79	88.89	94.44	88.46	98.08
haberman	Gm	57.01	62.43	61.57	63.65	67.96	65.83	62.36	69.13
	F	42.86	47.24	46.43	48.45	52.72	53.33	50.00	54.12
	AUC	62.08	62.88	63.23	64.10	68.10	68.33	66.32	69.17
Glass	Gm	79.96	77.17	79.95	69.30	69.82	81.65	80.14	81.65
	F	60.95	40.61	62.67	32.22	33.67	80.00	72.30	80.00
	AUC	81.28	77.82	81.28	70.64	71.82	83.33	80.13	83.33
Vehicle	Gm	96.64	94.98	98.14	96.63	96.64	97.94	98.71	98.71
	F	94.87	90.82	96.85	95.45	94.91	96.20	98.70	98.70
	AUC	96.66	94.99	98.14	96.68	96.66	97.94	98.72	98.72
Abalone	Gm	94.25	94.83	95.59	90.64	92.24	92.15	93.93	96.00
	F	84.49	84.84	85.55	78.62	79.27	82.62	81.25	86.67
	AUC	94.34	94.92	95.69	90.66	92.38	92.23	94.12	96.08
Ecoli	Gm	90.24	92.21	93.46	93.42	94.14	92.58	85.53	95.46
	F	75.00	77.03	83.67	82.83	81.15	73.68	82.22	85.72
	AUC	90.36	92.45	93.50	93.70	94.16	92.86	86.46	95.50
Balance	Gm	87.42	77.78	68.34	70.39	74.47	100.00	100.00	100.00
	F	77.78	28.46	33.58	26.81	28.29	100.00	100.00	100.00
	AUC	88.00	80.26	74.22	74.64	75.61	100.00	100.00	100.00
Libra	Gm	91.96	95.03	86.21	90.69	92.93	91.81	86.60	98.29
	F	91.43	62.44	79.28	82.22	87.30	89.21	85.71	93.02
	AUC	92.50	95.13	87.05	91.98	93.38	92.35	87.50	98.38
Yeast	Gm	60.95	70.11	68.42	54.89	69.74	60.45	60.89	65.51
	F	49.31	57.43	52.82	42.57	57.29	46.81	49.65	53.75
	AUC	65.30	70.14	67.28	62.04	70.00	62.87	65.61	67.71
Yeast1	Gm	71.44	84.83	83.16	80.68	83.04	77.81	78.83	84.95
	F	59.52	67.53	70.77	70.86	56.82	67.83	57.49	72.73
	AUC	73.82	85.03	84.04	82.11	83.19	79.99	80.82	85.61
Yeast2	Gm	84.95	89.43	88.80	88.96	88.75	89.57	90.38	93.83
	F	72.73	80.72	82.81	84.21	70.50	75.00	70.77	85.71
	AUC	85.61	89.81	89.28	89.46	88.95	89.73	90.43	93.91
Yeast3	Gm	91.93	84.02	84.02	80.65	81.02	89.44	89.56	93.31
	F	78.56	76.92	76.92	75.24	63.81	88.89	88.89	81.82
	AUC	92.04	85.13	85.13	82.51	82.28	90.00	90.00	93.37
Yeast4	Gm	83.01	81.54	81.76	83.06	82.09	78.78	78.78	83.30
	F	66.67	63.55	69.17	58.96	59.69	66.67	66.67	75.00
	AUC	83.95	83.09	83.37	83.24	83.17	80.89	80.89	84.15
Yeast5	Gm	49.73	68.07	65.04	64.92	60.27	61.14	53.27	68.76
	F	33.33	34.21	44.00	40.80	39.23	41.88	36.36	46.15
	AUC	61.96	70.31	70.77	70.60	67.91	68.53	63.94	70.91
Block	Gm	90.86	94.06	93.08	93.76	94.23	89.03	87.32	94.73
	F	83.07	82.87	83.26	79.96	81.02	80.18	80.37	84.96
	AUC	91.12	94.11	93.18	93.87	94.05	89.40	87.92	94.94
Block1	Gm	93.28	93.78	94.05	93.47	92.66	89.73	92.41	93.09
	F	87.60	79.15	77.69	81.23	77.79	79.17	86.15	87.64
	AUC	93.46	93.88	94.13	93.59	92.87	90.12	92.64	93.28
Block2	Gm	80.43	93.44	89.57	93.33	93.71	87.82	85.80	93.06
	F	65.22	65.29	64.58	65.98	63.20	63.34	80.95	65.37
	AUC	82.20	93.48	89.97	93.40	93.72	89.59	86.75	93.09
Block3	Gm	85.67	87.69	83.62	82.98	81.78	74.72	71.97	89.11
	F	78.16	50.19	49.22	53.99	53.39	50.04	57.14	50.06
	AUC	86.60	88.30	84.59	84.13	83.21	84.40	75.73	89.43

## Data Availability

Not applicable.
